# Pulsations as a Signal of Danger: A Case of Scalp Cirsoid Aneurysm

**DOI:** 10.7759/cureus.63333

**Published:** 2024-06-27

**Authors:** Charusheela R Gore, Padmakar Bardapurkar, Aakriti Kundlia, Sushama G Gurwale

**Affiliations:** 1 Department of Pathology, Dr. D. Y. Patil Medical College, Hospital and Research Centre, Dr. D. Y. Patil Vidyapeeth, Pune (Deemed to be University), Pune, IND

**Keywords:** endovascular embolization, pulsatile mass, vascular anomalies, vessels, arteriovenous malformations (avms), cirsoid aneurysm

## Abstract

Cirsoid aneurysms, formerly identified as arteriovenous malformations (AVMs), represent infrequent vascular anomalies primarily localized within the scalp. These anomalies are typified by the absence of interposing capillaries, giving rise to extensively vascularized, expanded conduits connecting arterial feeders and venous outflows. This report details a case of a 13-year-old male afflicted with a cirsoid aneurysm in the scalp, who presented with swelling on the left frontal region, accompanied by headache and pulsatile sensations. Definitive diagnosis was achieved through radiological and histopathological examinations. Scalp cirsoid aneurysms may either be congenital in nature or arise following traumatic incidents, with clinical manifestations typically surfacing in the third decade of life. Common clinical presentations encompass a palpable, pulsatile subcutaneous mass, throbbing headaches, tinnitus, and cosmetic concerns. Diverse therapeutic strategies, including surgical excision, endovascular embolization, and percutaneous injection of sclerosing agents, can be employed contingent upon the particular characteristics of the lesion.

## Introduction

The term "cirsoid," introduced by Brecht in 1833 and rooted in the Greek word "kirsos," meaning "varice," emerged in 19th-century scientific literature [1]. Cirsoid aneurysms, previously known by various names including arteriovenous malformations (AVMs), arteriovenous fistula, aneurysm serpentinum, aneurysm racemosum, and plexiform angioma, are rare vascular anomalies [2-4]. Reports suggest that cirsoid aneurysms constitute 8.1% of all AVM cases [3]. Scalp cirsoid aneurysms, an infrequent condition, occur within the vascular system, connecting feeding arteries to drainage veins without capillaries [4]. This unique feature, combined with numerous endothelial-lined channels, makes these lesions highly vascular [5]. The feeding arteries mainly originate from vessels that supply the scalp, such as the external carotid, supraorbital, occipital, and superficial temporal arteries. These arteries converge into dilated veins in the scalp or facial regions, resulting in various degrees of cosmetic abnormalities [5]. In our study, we present a case of a scalp cirsoid aneurysm, characteristically presenting with left frontal swelling associated with headache and pulsations.

## Case presentation

**Figure 1:**
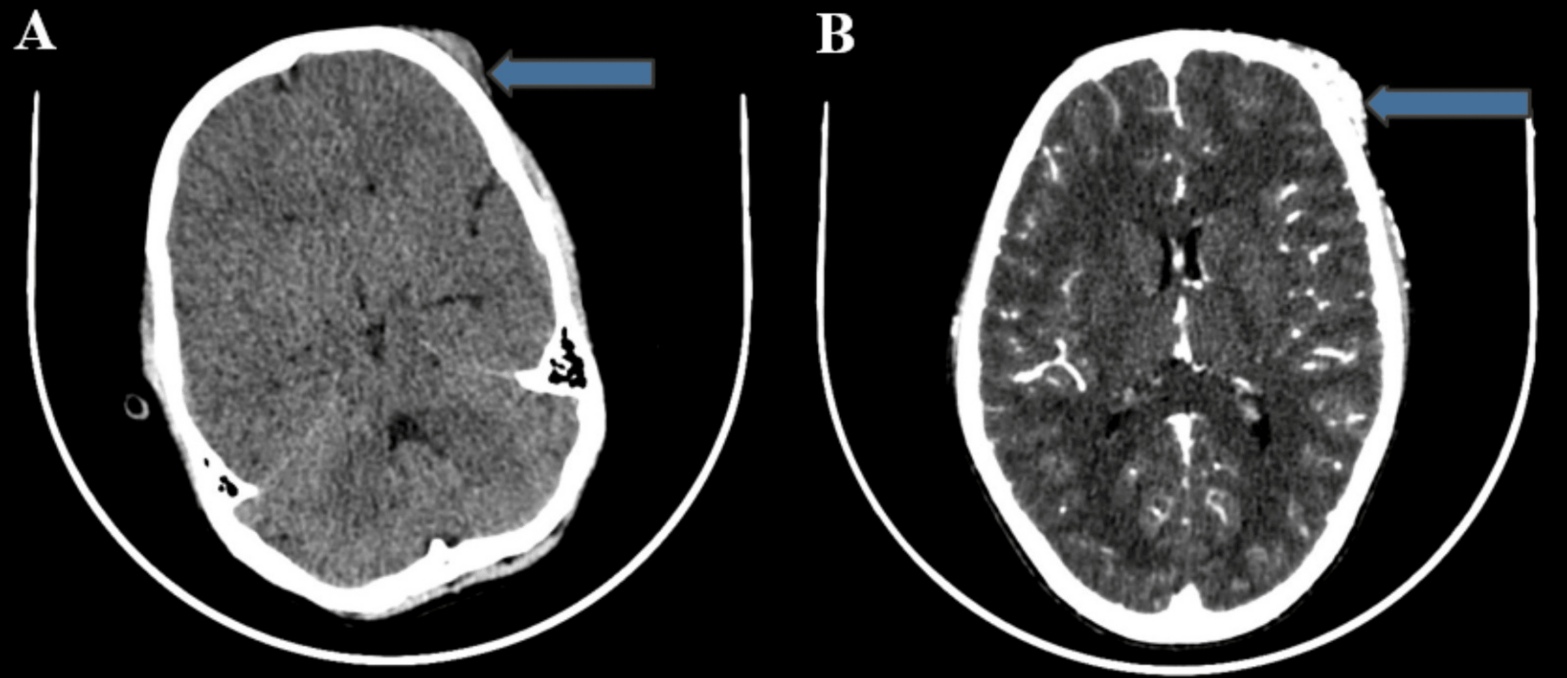
(A) CT brain (plain) and (B) CT carotid angiography showing a soft tissue density lesion in the subcutaneous plane of the scalp in the left frontal region with significant enhancement (blue arrow). This finding is suggestive of an arteriovenous malformation.

A 13-year-old male patient presented with a persistent left frontal swelling that had developed gradually over four years, accompanied by headaches and pulsations. He had no history of trauma, pain, neurological symptoms, or chronic health conditions. On examination, a 4 x 4 cm pulsatile mass was observed on the left forehead. CT scans and angiography revealed a subcutaneous scalp lesion with significant enhancement, confirming an AVM (Figure 1).

**Figure 2:**
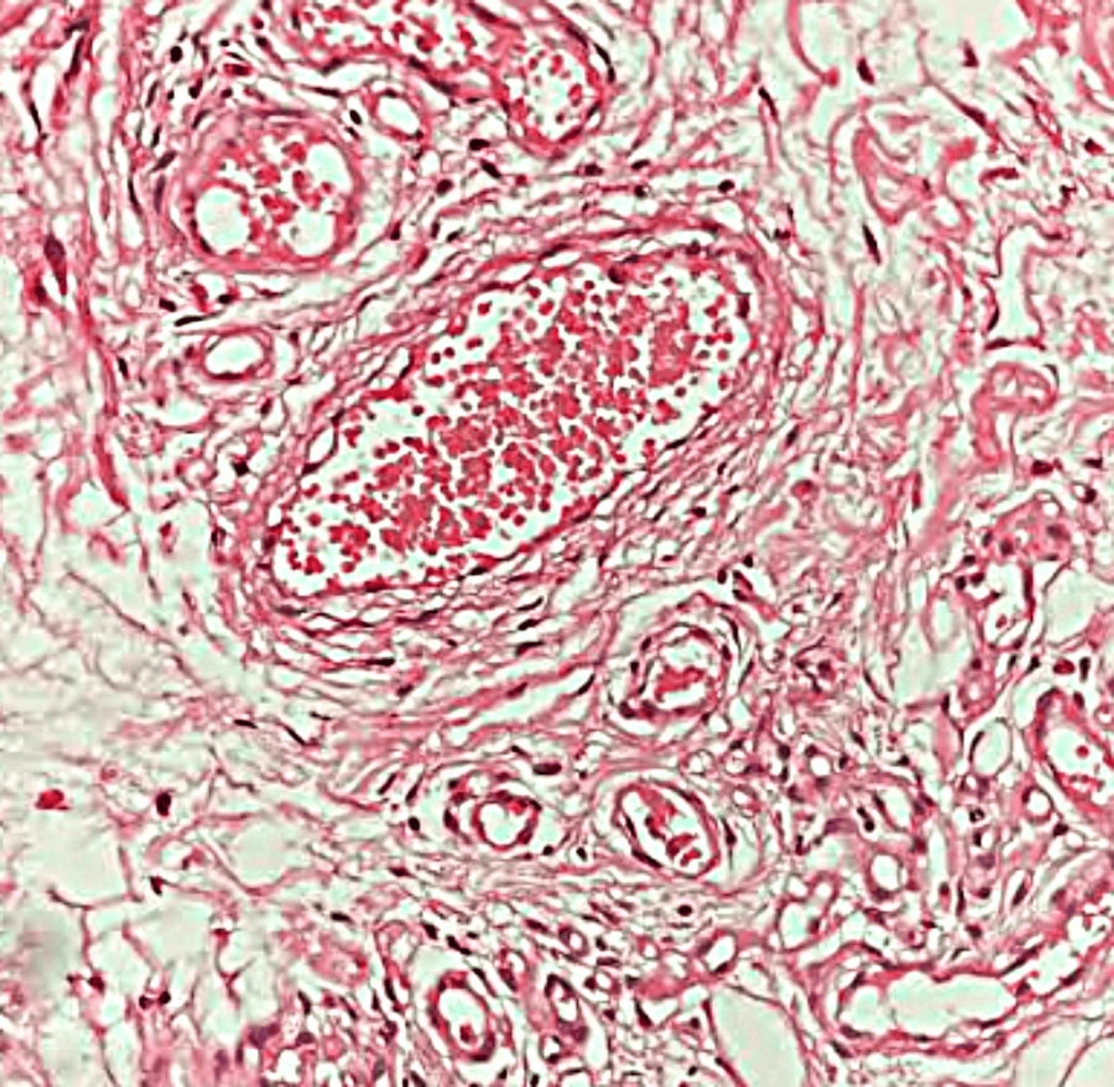
Loose fibro-collagenous and fibrofatty tissue with proliferation of vessels of varying size and caliber. Few vessels are dilated and congested. Occasional hypertrophied nerve bundles are observed (hematoxylin and eosin, x200).

Biopsy showed gray-brown, firm tissue with vessels of varying sizes, some dilated and congested, and hypertrophied nerve bundles. This confirmed a diagnosis of scalp AVM, consistent with a cirsoid aneurysm (Figure 2).

## Discussion

Scalp AVMs primarily occur in the periosteal layer beneath the galea or within the temporal fascia, evenly distributed across the frontal, parietal, and temporal regions [5]. These AVMs can stem from diverse factors, including congenital origins, trauma, idiopathic causes, previous craniotomy procedures, implantation, hair infections, and inflammation [3,5]. Congenital AVMs are more common than traumatic cirsoid aneurysms, especially among pediatric patients, where they are thought to be present since birth [5]. Traumatic AVMs may result from penetrating or blunt trauma, prior surgical interventions, or hair transplantation [3,5]. They may manifest months or years after a scalp injury, forming a fistulous connection due to mechanical damage to arterial and venous walls. These AVMs can develop into high-flow arterial-to-venous shunts or occur through thrombus canalization [5].

Typically, congenital scalp cirsoid aneurysms become symptomatic in the third decade of life, more frequently affecting males [4]. These lesions often present as subcutaneous pulsatile masses, occasionally initially misdiagnosed as benign scalp irregularities [4]. Usually, the lesion starts as a tiny subcutaneous lump on the head and grows into a large, deforming mass [1]. Symptoms can vary depending on the AVM's size, progressing from a small subcutaneous lump to a prominent, deforming head mass [5]. Common symptoms include murmurs, headaches, bleeding, tinnitus, paresthesia, local pain, burning sensations, scalp necrosis, local bleeding, aesthetic concerns, ulceration, and hemorrhage of the nidus and, in severe cases, congestive heart failure [1,3,5,6]. Hemorrhage serves as the primary symptom in up to 11% of individuals with scalp AVMs [5]. Although these lesions are benign and extracranial, in rare cases, they can exert pressure on the brain, causing neurological deficits or seizures [5]. Improper redirection of common carotid blood flow can lead to conditions like epilepsy, mental retardation, and cerebral ischemia [3,5]. Hormonal changes and pregnancy can exacerbate symptoms associated with scalp AVMs [5].

The primary blood supply to scalp cirsoid aneurysms comes from the superficial temporal artery, as in our case, followed by the occipital artery. The superficial temporal artery is particularly vulnerable as it lacks protective muscle fibers when crossing the superior temporal line [5]. Matsushige et al. [7] described three stages in the formation of scalp AVMs: in the initial stage, single or multiple feeders from extracranial branches of the extracranial carotid arteries (ECAs) supply the lesion; in the second stage, additional feeders arise from ECA's intracranial branches, traversing the skull bone; and in the third stage, multiple feeders originate from ECA, meningeal, and pial arteries. This staging system aids in selecting suitable surgical treatment plans based on angiographic features. Shenoy et al. [8] categorized scalp abnormalities into two groups based on the origin of feeding arteries. Group I includes primary scalp AVMs with venous drainage into the scalp venous system and arterial feeders from the calvarial branches of the external carotid, ophthalmic, and vertebral arteries. Group II pertains to primary outflow vessels of cerebral vascular abnormalities characterized by dilated channels. While major scalp vascular abnormalities can be safely removed, addressing secondary scalp venous dilatation without treating the cerebral component can be risky. Yokouchi et al. [9] classified AVMs into three groups based on their anomalous connections. Additionally, Gopinath et al. [10] classified scalp AVMs into two types: plexiform and fistulous, depending on their angiographic features. The plexiform variant presents a more complex network of aberrant vessels within the nidus, while the fistulous type features an aberrant arterial feeder directly emptying into a venous reservoir without intricate tangles.

To differentiate vascular lesions such as cavernous hemangioma, venous malformation, sinus pericranii, and aneurysms, performing selective angiography is essential [11]. Digital subtraction angiography serves as the gold standard for diagnosis due to its ability to precisely visualize vascular anatomy and flow dynamics. However, non-invasive modalities like CT or magnetic resonance imaging (MRI) are also valuable. They provide detailed information regarding the location, extent, and configuration of scalp fistulas, offering complementary insights alongside angiography. This integrated approach aids in accurate diagnosis and treatment planning for these complex vascular conditions [4]. Due to their complex vascular anatomy, intracranial anastomoses, high-flow bypasses, and potential cosmetic concerns, treating scalp AVMs can be challenging and must be chosen carefully [1]. Treatment decisions should consider clinical features, lesion morphology, the patient's age, lesion size, location, and Schobinger stage [5]. A multidisciplinary approach is often used, involving surgical excision, as in our case, endovascular embolization, direct puncture embolization, percutaneous injection of sclerosing drugs, or a combination of these methods. In cases of abnormal vascular circulation, surgical resection of arteriovenous fistulae is frequently employed for a permanent solution [4].

## Conclusions

Scalp cirsoid aneurysms, previously known as AVMs, represent uncommon yet significant vascular irregularities primarily localized within the scalp. They can manifest either congenitally or as a consequence of trauma, giving rise to a spectrum of distressing clinical symptoms. Timely and accurate diagnosis, relying on clinical manifestations, imaging studies, and histopathological assessments, is imperative for determining the most suitable treatment approach. The management of these anomalies often necessitates a multidisciplinary effort, taking into account the patient's age, lesion size, location, and developmental stage. Various treatment modalities, including surgical resection and endovascular interventions, have proven effective in addressing scalp cirsoid aneurysms, providing relief from symptoms and mitigating the risk of potential complications. The challenges posed by these lesions underscore the importance of a comprehensive understanding of vascular anomalies and the requirement for tailored strategies to address these distinctive clinical scenarios.
